# Elevated extracellular matrix production and degradation upon bone morphogenetic protein-2 (BMP-2) stimulation point toward a role for BMP-2 in cartilage repair and remodeling

**DOI:** 10.1186/ar2305

**Published:** 2007-10-08

**Authors:** Esmeralda N Blaney Davidson, Elly L Vitters, Peter LEM van Lent, Fons AJ van de Loo, Wim B van den Berg, Peter M van der Kraan

**Affiliations:** 1 Experimental Rheumatology and Advanced Therapeutics, Radboud University Nijmegen Medical Centre, Geert Grooteplein 26-28, Nijmegen, 6500 HB, The Netherlands

## Abstract

Bone morphogenetic protein-2 (BMP-2) has been proposed as a tool for cartilage repair and as a stimulant of chondrogenesis. In healthy cartilage, BMP-2 is hardly present, whereas it is highly expressed during osteoarthritis. To assess its function in cartilage, BMP-2 was overexpressed in healthy murine knee joints and the effects on proteoglycan (PG) synthesis and degradation were evaluated. Moreover, the contribution of BMP in repairing damage induced by interleukin-1 (IL-1) was investigated. Ad-BMP-2 was injected intra-articularly into murine knee joints, which were isolated 3, 7, and 21 days after injection for histology, immunohistochemistry, and autoradiography. In addition, patellar and tibial cartilage was isolated for RNA isolation or measurement of PG synthesis by means of ^35^SO_4 _^2- ^incorporation. To investigate the role for BMP-2 in cartilage repair, cartilage damage was induced by intra-articular injection of IL-1. After 2 days, Ad-BMP-2, Ad-BMP-2 + Ad-gremlin, Ad-gremlin, or a control virus was injected. Whole knee joints were isolated for histology at day 4 or patellae were isolated to measure ^35^SO_4_^2- ^incorporation. BMP-2 stimulated PG synthesis in patellar cartilage on all days and in tibial cartilage on day 21. Aggrecan mRNA expression had increased on all days in patellar cartilage, with the highest increase on day 7. Collagen type II expression showed a similar expression pattern. In tibial cartilage, collagen type II and aggrecan mRNA expression had increased on days 7 and 21. BMP-2 overexpression also induced increased aggrecan degradation in cartilage. VDIPEN staining (indicating matrix metalloproteinase activity) was elevated on day 3 in tibial cartilage and on days 3 and 7 in patellar cartilage, but no longer was by day 21. Increased NITEGE staining (indicating aggrecanase activity) was found on days 7 and 21. In IL-1-damaged patellar cartilage, BMP-2 boosted PG synthesis. Blocking of BMP activity resulted in a decreased PG synthesis compared with IL-1 alone. This decreased PG synthesis was associated with PG depletion in the cartilage. These data show that BMP-2 boosts matrix turnover in intact and IL-damaged cartilage. Moreover, BMP contributes to the intrinsic repair capacity of damaged cartilage. Increased matrix turnover might be functional in replacing matrix molecules in the repair of a damaged cartilage matrix.

## Introduction

Cartilage damage is a major problem in joint diseases like osteoarthritis (OA) and rheumatoid arthritis. As a response to cartilage injury, chondrocytes display a reparative response [1,2]. Unfortunately, this response is very limited, resulting in suboptimal repair [3]. Until now, reparative responses that have been induced by drilling and microfractures have been unable to overcome this problem [4]. They yield a new tissue, often fibrocartilage that does not compare to original cartilage in structural, biomechanical, and biochemical aspects [5]. Currently, in experimental settings, growth factors are used to promote chondrogenic differentiation *in vitro*. This has the potential to eventually produce cartilage that can overcome the current problems.

Bone morphogenetic protein-2 (BMP-2) is one of the candidate growth factors with good potential in cartilage tissue engineering as well as cartilage repair. BMP-2 belongs to the transforming growth factor-beta (TGF-β) superfamily, consisting of TGF-βs, growth differentiation factors, BMPs, activins, inhibins, and glial cell line-derived neurotrophic factor [6]. BMPs have been identified as very potent inducers of bone, but since then it has become evident that their function is not limited to skeletal development [7]. BMP-2 expression is found in mesenchymal condensation in embryonic development [8]. BMP-2 is able to induce chondrogenesis in human mesenchymal stem cells in culture [9]. For cartilage reparative reasons, BMP-2 can be used to induce chondrogenesis by coating a scaffold with BMP-2 before implantation [10]. Thereby, the scaffold itself can be replaced by the original tissue. This can be combined with culturing mesenchymal stem cells or tissue-specific cells on the coated scaffold to gain *de novo *tissue formation in the scaffold [11]. Although BMP-2 is able to induce cartilage formation, we found that the expression of BMP-2 in healthy cartilage was low but that its expression was elevated in areas surrounding cartilage lesions and in OA cartilage [12]. In addition, mechanical injury was found to upregulate BMP-2 as well as BMP-2 signalling in human cartilage explants [13]. This could indicate that BMP-2 is upregulated as a reparative response but could also indicate that BMP-2 is merely upregulated as a pathological side effect, thereby further stimulating injury. Therefore, the effect of elevated BMP-2 on healthy cartilage and in cartilage that has been damaged by exposure to interleukin-1 (IL-1) was investigated.

## Materials and methods

### Construction of the BMP-2 adenovirus

A polymerase chain reaction (PCR) was performed on cDNA of synovial fibroblast cells isolated from human knee joint biopsy samples. As primers (Biolegio, Nijmegen, The Netherlands), 5'-CCCAGCGTGAAAGAGAGAC-3' (forward primer) and 5'-AAATCTAGACTAGCGA-3' (reverse primer) were used, thereby introducing the XbaI restriction site. The PCR product was ligated blunt into the Srf restriction site of the PCR-Script vector (Stratagene, La Jolla, CA, USA). The vector containing the product was introduced into JM109 cells via heat shock and plated on ampicilin-resistant agar plates. Several colonies were cultured and the vector was isolated by miniprep (QIAGEN, Venlo, The Netherlands) according to manufacturer protocol, followed by restriction analysis. The miniprep product of one of the colonies that contained the BMP-2 PCR product was cut with restriction enzymes XbaI and SalI (New England Biolabs, Inc., Ipswich, MA, USA). The same restriction was performed on the pShuttle-CMV vector (Stratagene). Thereafter, the PCR product that was isolated from the PCR-Script vector was ligated into the pShuttle-CMV vector using T4 DNA Ligase (Invitrogen Corporation, Carlsbad, CA, USA). The adenovirus was then produced with the AdEasy Adenoviral Vector System (Stratagene) by co-transfection of the vector with the plasmid in N52E6 cells according to manufacturer protocol.

### Construction of the gremlin adenovirus

An adenovirus overexpressing the BMP-inhibitor gremlin was constructed. Therefore, a PCR was performed on cDNA of 3T3 cells. The following primers were used: 5'-ACCACCATGAATCGCACCGC-3' (forward primer) and 5'-GTCAAAGCGGGCACATTCA-3' (reverse primer) (Biolegio). The PCR product was ligated blunt into the Srf restriction site of the PCR-Script vector (Stratagene). The vector containing the product was introduced into JM109 cells via heat shock and plated on ampicilin-resistant agar plates. Several colonies were cultured and the vector was isolated by miniprep (QIAGEN) according to manufacturer protocol, followed by restriction analysis. The miniprep product of one of the colonies that contained the gremlin PCR product was used for PCR again in order to introduce the XhoI and XbaI restriction sites. This was performed with 5'-CCGCTCGAGACCACCATGAATCGCACCGC-3' as a forward primer and 5'-GCTCTAGATGAATGTGCCCGCTTGAC-3' as the reverse primer. The PCR product was cut with XhoI and XbaI. The same enzymes were used to cut the pShuttle-CMV vector (Stratagene). The PCR product was then ligated into the pShuttle-CMV vector using T4 DNA Ligase (Invitrogen Corporation). The adenovirus was produced with the AdEasy Adenoviral Vector System (Stratagene) by co-transfection of the vector with the plasmid in N52E6 cells according to manufacturer protocol.

### Functional test Ad-BMP-2 and Ad-gremlin: BRE-luciferase stably transfected cell line

The BMP-responsive element (BRE)-luciferase construct was obtained from Peter ten Dijke [14]. It contains a BRE that drives a luciferase gene. The BRE-luciferase construct was isolated from its pGL3-Basic vector by cutting with the enzymes MluI and BamHI (New England Biolabs, Inc.). The pcDNA3.1(-)/Myc-HisB vector (Invitrogen Corporation) was cut with the same enzymes, thereby also removing the CMV promoter. Subsequently, the BRE-luciferase construct was cloned into the pcDNA3.1 vector. After restriction analysis confirming the correct product, 3T3 cells were transfected with polyfectamin (Invitrogen Corporation) and cultured with neomycin (800 μg/mL). By limiting dilution cloning, a cell line was created. Its responsiveness was effectively tested with serial dilutions of BMP-2 (R&D Systems, Inc., Minneapolis, MN, USA) in culture medium as well as a combination of BMP-2 with several concentrations of noggin (R&D Systems, Inc.).

To test the functionality of the BMP-2 adenovirus, 911 cells were transfected with Ad-BMP-2 (multiplicity of infection [MOI] 10). After 48 hours, the supernatant of the cells was incubated with the BRE-luciferase cell line. The luciferase production had reached a maximum, thus production could not be quantified. Therefore, the supernatant of the transfected cells was diluted 25 or 125 times to measure the quantity of BMP-2 that was produced.

To test the functionality of the Ad-gremlin adenovirus, 3T3 cells were transfected with Ad-gremlin (MOI 25). After 28 hours, the supernatant of the cells was incubated with the BRE-luciferase cell line and a variety of known concentrations of BMP-2 protein.

### Animals

Eight-week-old male C57Bl/6N mice (*n *= 272) were used. Mice were kept in filter-top cages with woodchip bedding under standard pathogen-free conditions. They were fed standard diet and tap water *ad libitum*. This study has been approved by the local animal experimentation committee, Nijmegen, The Netherlands.

### Experimental design

To assess the effect of BMP-2 on healthy cartilage, mice were injected intra-articularly with either Ad-BMP-2 (an adenovirus expressing human BMP-2) or Ad-luc as a control virus (an adenovirus expressing the luciferase gene) at a plaque-forming unit (PFU) count of 2 × 10^6^. After 3, 7, and 21 days, knee joints were isolated for histology, autoradiography, and immunohistochemistry (*n *= 30; 5 mice per group per time point), for RNA isolation of tibial and patellar cartilage (*n *= 54; 9 mice per group per time point), or for measurement of proteoglycan synthesis by ^35^SO_4_^2- ^incorporation in tibial and patellar cartilage (*n *= 72; 12 mice per group per time point).

In addition, the role of BMP in the intrinsic cartilage repair upon damage was investigated. Therefore, mice were injected with 6 μL of solution of IL-1β (10 ng/knee) (R&D Systems, Inc.) in 0.9% NaCl intra-articularly into the right knee joint (*n *= 116). Two days after IL-1 injection, Ad-BMP-2 (PFU of 2 × 10^6^), an adenovirus expressing the specific BMP-inhibitor gremlin (Ad-gremlin; PFU of 1 × 10^7^), a combination of both, or a control virus (Ad-luc) that has been previously described [15] was injected intra-articularly into the right knee joint (PFU of 1 × 10^7^). Four days after IL-1 injection, patellae were isolated for proteoglycan synthesis measurement by ^35^SO_4_^2- ^incorporation (*n *= 92) or whole knee joints were isolated for histological assessment of proteoglycan content of patellar and tibial cartilage (*n *= 24). Gremlin inhibits not only signalling of BMP-2, but also that of other BMPs. Therefore, in cases in which gremlin was used, BMP instead of BMP-2 was mentioned.

### Histology

Knee joints of mice were isolated and fixed for 7 days in phosphate-buffered formalin. They were decalcified for a week in 10% formic acid. Knee joints were dehydrated with an automated tissue processing apparatus (Miles Scientific Tissue-Tek VIP tissue processor; Miles Scientific, now part of Bayer Corp., Emeryville, CA, USA) and embedded in paraffin. Frontal whole sections of 7 μm were made. Sections were used for immunohistochemistry, autoradiography, or stained with safranin O and counterstained with fast green (Brunschwig chemie, Amsterdam, The Netherlands).

### Quantitative PCR

Patellar and tibial cartilage was stripped off the joint as previously described [16] (time points 3, 7, and 21 after injection of the adenovirus; *n *= 9 per group per time point). RNA was isolated from the tissue with an RNeasy Mini Kit (QIAGEN) after which a reverse transcription-PCR was performed. Individual samples of each group were pooled, and a quantitative PCR (Q-PCR) was run in duplicate. A Q-PCR was prepared as follows: a primer mix of 1.5 μL of forward primer (5 μM), 1.5 μL of reverse primer (5 μM), and 4.5 μL of dH_2_O was added to 12.5 μL of Sybr Green PCR master mix (Applied Biosystems, Foster City, CA, USA). Then, 5 μL of cDNA was added and the Q-PCR was performed by an ABI/PRISM 7000 sequence detection system (Applied Biosystems) according to manufacturer protocol. PCR conditions were 2 minutes at 50°C and 10 minutes at 95°C followed by 40 cycles of 15 seconds at 95°C and 1 minute at 60°C, with data collection in the last 30 seconds. In addition, for each PCR, melting curves were run. The genes that were measured and the corresponding primer sets are presented in Table 1. Efficiencies for all primer sets were determined (Table 1) using a standard curve of five serial cDNA dilutions in water in duplicate. Primers were accepted if the deviation from the slope of the standard curve was less than 0.3 compared with the slope of the *GAPDH *standard curve and if the melting curve showed only one product. For each primer pair, non-template controls were run in duplicate. The cycle threshold (Ct) values of the genes of interest were corrected for the Ct of the reference gene *GAPDH*. Relative mRNA expression was calculated by 2 to the power of delta Ct. Gene expression levels after transfection with BMP-2 were compared with the control virus group. If the mRNA expression was higher after BMP-2 expression, the fold change is positive and decreases in expression are negative.

**Table 1:** Murine primers used for quantitative polymerase chain reaction

Gene	*R*^2^	Efficiencies	Forward primer (5'→3')	Reverse primer (5'→3')
*GAPDH*	0.997	2.05	GGCAAATTCAACGGCACA	GTTAGTGGGGTCTCGCTCCTG
Collagen IA	0.997	2.10	TGACTGGAAGAGCGGAGAGTACT	CCTTGATGGCGTCCAGGTT
Collagen II	0.992	2.15	TTCCACTTCAGCTATGGCGA	GACGTTAGCGGTGTTGGGAG
Collagen III	0.997	2.05	CCCCGAGGGCTGTGCTA	TGAACTTCAACTGGAACAGGGTATC
Aggrecan	0.992	2.15	TCTACCCCAACCAAACCGG	AGGCATGGTGCTTTGACAGTG
Collagen X	0.992	1.97	CACACTCTGTCCTCGTGCTTTG	GGAATCCCTGTAAGACACACCAA

### Quantitative measurement of proteoglycan synthesis

Proteoglycan synthesis was assessed by measurement of ^35^SO_4_^2- ^incorporation. Isolated patellae and tibia were immediately placed in Dulbecco's modified Eagle's medium (Invitrogen Corporation) with gentamicin (Centrafarm Services B.V., Etten-Leur, The Netherlands) (50 mg/mL) and pyruvate (Invitrogen Corporation). After half an hour, medium was replaced by medium containing ^35^SO_4_^2- ^(20 μCi/mL) and incubated for 3 hours at 37°C 5% CO_2_. Thereafter, patellae and tibia were further prepared for determining the amount of ^35^SO_4_^2- ^incorporation in the articular cartilage as previously described using a liquid scintillation counter [17]. Cartilage from the separate surfaces of one tibia was pooled.

### Autoradiography

For assessment of proteoglycan synthesis, the amount of ^35^SO_4_^2- ^incorporation in cartilage was measured histologically. Mice were injected intraperitoneally with 75 μCi radiolabelled ^35^SO_4_^2- ^4 hours prior to knee joint isolation. After histological processing, sections were dipped in nuclear research emulsion (Ilford, Basildon, Essex, UK) and exposed for 4 to 8 weeks. Slides were developed in Kodak D-19 developer (Kodak, Chalon-sur-Saone, France) and counterstained with hematoxylin and eosin.

### Immunohistochemistry: NITEGE

Sections were deparaffinized and washed with phosphate-buffered saline (PBS). Sections were incubated in citrate buffer (0.1 M sodium citrate + 0.1 M citric acid) for 2 hours for antigen unmasking. Endogenous peroxidase was blocked with 1% hydrogen peroxide in methanol for 30 minutes. Sections were blocked with 5% normal serum of the species in which the secondary antibody was produced. Specific primary antibodies were incubated overnight at 4°C. To assess degradation of aggrecan, a polyclonal antibody to the aggrecan neoepitope NITEGE (1:1,000) (Aggrecan Neo) (Acris, Hiddenhausen, Germany) was used. The antibody recognizes CGGNITEGE, which is an epitope revealed from aggrecan core proteins upon aggrecanase cleavage at the Glu373-Ala374 site. After extensive washing with PBS, the appropriate biotin-labeled secondary antibody was used (DAKO Denmark A/S., Glostrup, Denmark) for 30 minutes at room temperature followed by a biotin-streptavidin detection system according to manufacturer protocol (Vector Laboratories, Burlingame, CA, USA). Bound complexes were visualized using diaminobenzidine reagent (Sigma-Aldrich, St. Louis, MO, USA), counterstained with hematoxylin (Merck & Co., Inc., Whitehouse Station, NJ, USA), dehydrated, and mounted with Permount (Fischer Scientific, New Jersey, USA).

### Immunohistochemistry: VDIPEN staining

After deparaffinization of the sections, they were digested with chondroitinase ABC for 2 hours at 37°C. Then the sections were treated with 1% H_2_O_2 _in methanol for 20 minutes and subsequently washed with 0.1% Triton X-100 in PBS for 5 minutes followed by an incubation in 1.5% normal goat serum for 20 minutes. The primary antibody was affinity-purified rabbit anti-VDIPEN immunoglobulin G, detecting the VDIPEN C-terminal neoepitope of aggrecan generated by matrix metalloproteinases (MMPs) [18-20]. The primary antibody was incubated overnight at room temperature. As a secondary antibody, biotinylated goat anti-rabbit antibody was used and detected with biotin-streptavidin-peroxidase staining (Elite kit; Vector Laboratories). Peroxidase staining was developed using nickel enhancement and counterstained with orange G (2%).

### Histological scores

A blinded observer scored sections stained with safranin O, VDIPEN, NITEGE, and autoradiography. The uncalcified area of the cartilage surfaces was selected in at least three sections per knee joint. The computerized imaging system subsequently determined the area that stained positive and the total area that was selected. The percentage of the total area that stained positive was calculated. A computerized imaging system was used for all histological measurements (Qwin; Leica Imaging Systems Ltd., Wetzlar, Germany). The obtained values were averaged per knee joint.

### Statistical analysis

Data were analyzed with a Student *t *test. *P *values of less than 0.05 were considered significant. Error bars in all graphs display the standard error of the mean. Bonferroni correction was performed in cases of multiple comparisons.

## Results

### Ad-BMP-2 and Ad-gremlin tested on stably transfected BRE-luciferase cell line

To examine the efficiency of Ad-BMP-2, 911 cells were transfected with Ad-BMP-2 at an MOI of 10. The amounts of BMP-2 produced in the diluted samples were 40.16 ng/mL in the sample diluted 25 times and 8.39 ng/mL in the sample diluted 125 times. Thus, transfection with MOI 10 Ad-BMP-2 results in a production of 1 μg/mL BMP-2 biologically active protein after 48 hours.

Co-incubation of several dilutions of BMP-2 protein with the supernatant of cells transfected with Ad-gremlin showed that gremlin was able to effectively block luciferase expression whereas supernatant of control virus transfected cells had no effect. Thus, transfection with the Ad-gremlin adenovirus results in efficient blocking of BMP-2.

### Histological appearance of cartilage

**Figure 1:**
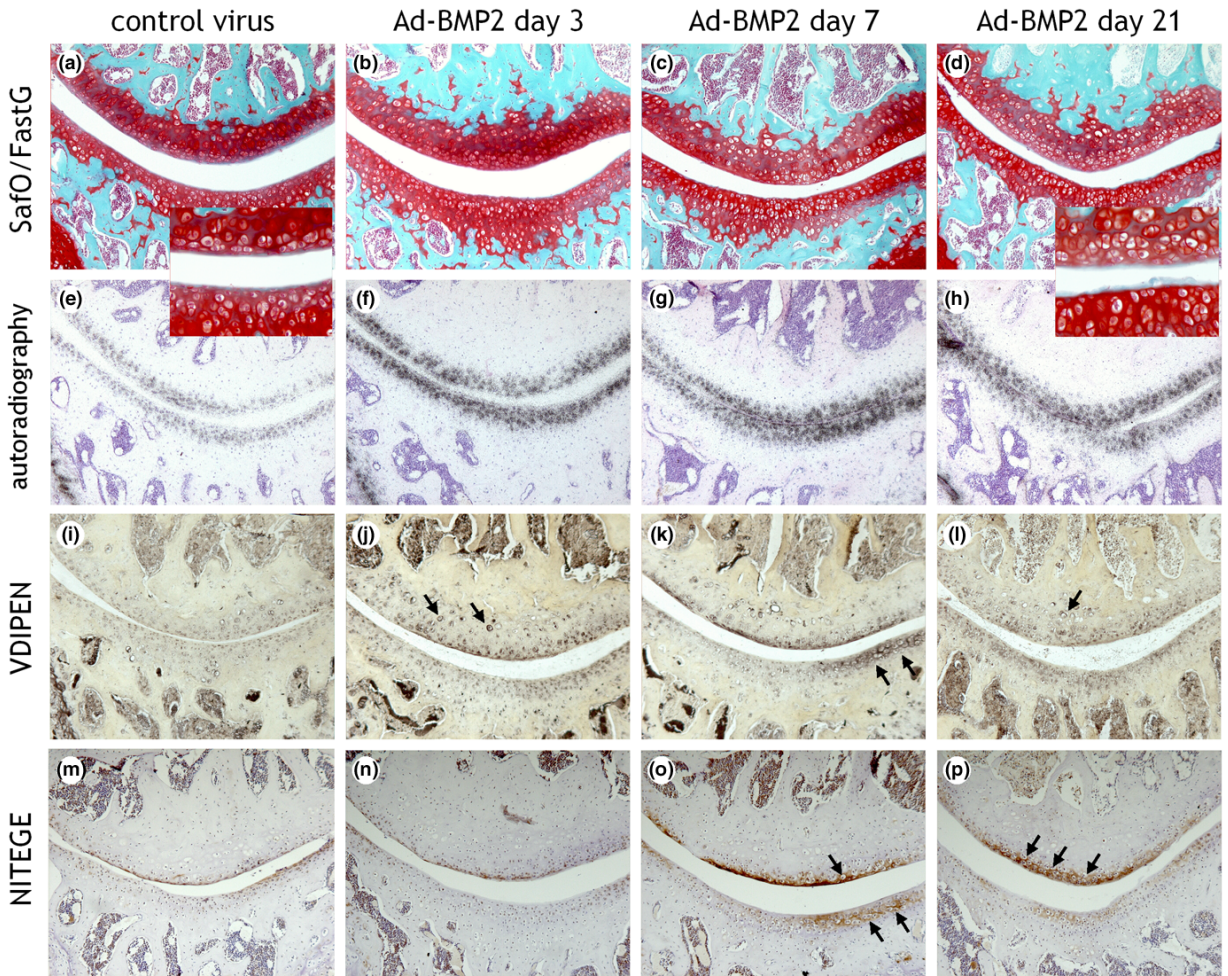
Histological appearance of knee joints injected with an adenovirus overexpressing bone morphogenetic protein-2 (BMP-2). Right knee joints were injected intra-articularly with Ad-BMP-2 or a control virus. Mice were injected with ^35^SO_4_^2- ^prior to knee joint isolation for histology on days 3, 7, or 21. Paraffin sections were stained with safranin O/fast green **(a-d)**, prepared for autoradiography **(e-h)**, and stained immunohistochemically for VDIPEN **(i-l) **or NITEGE **(m-p)**. Controls displayed here are from day 3 **(a,e,i,m)**. Cartilage of mice injected with Ad-BMP-2 appeared to have larger chondrocytes than controls **(c,d)**. Proteoglycan synthesis had increased by stimulation with BMP-2 **(f-h)**. BMP-2 stimulation also leads to increased VDIPEN staining **(k) **and NITEGE staining **(q,p)**. Arrows point to intense staining around chondrocytes. FastG, fast green; SafO, safranin O.

To assess the effect of BMP-2 overexpression on joint cartilage, C57Bl/6N mice were injected with either Ad-BMP-2 or Ad-luc. BMP-2 overexpression resulted in an altered appearance of chondrocytes in the cartilage. The chondrocytes that had been exposed to BMP-2 were larger than normal. In some joints, this was already visible by day 3, but all joints displayed altered chondrocyte appearance by day 7 (Figure 1a–d). This was more apparent in the patella than in the tibia.

### BMP-2 induces expression of aggrecan and collagen type II

**Figure 2:**
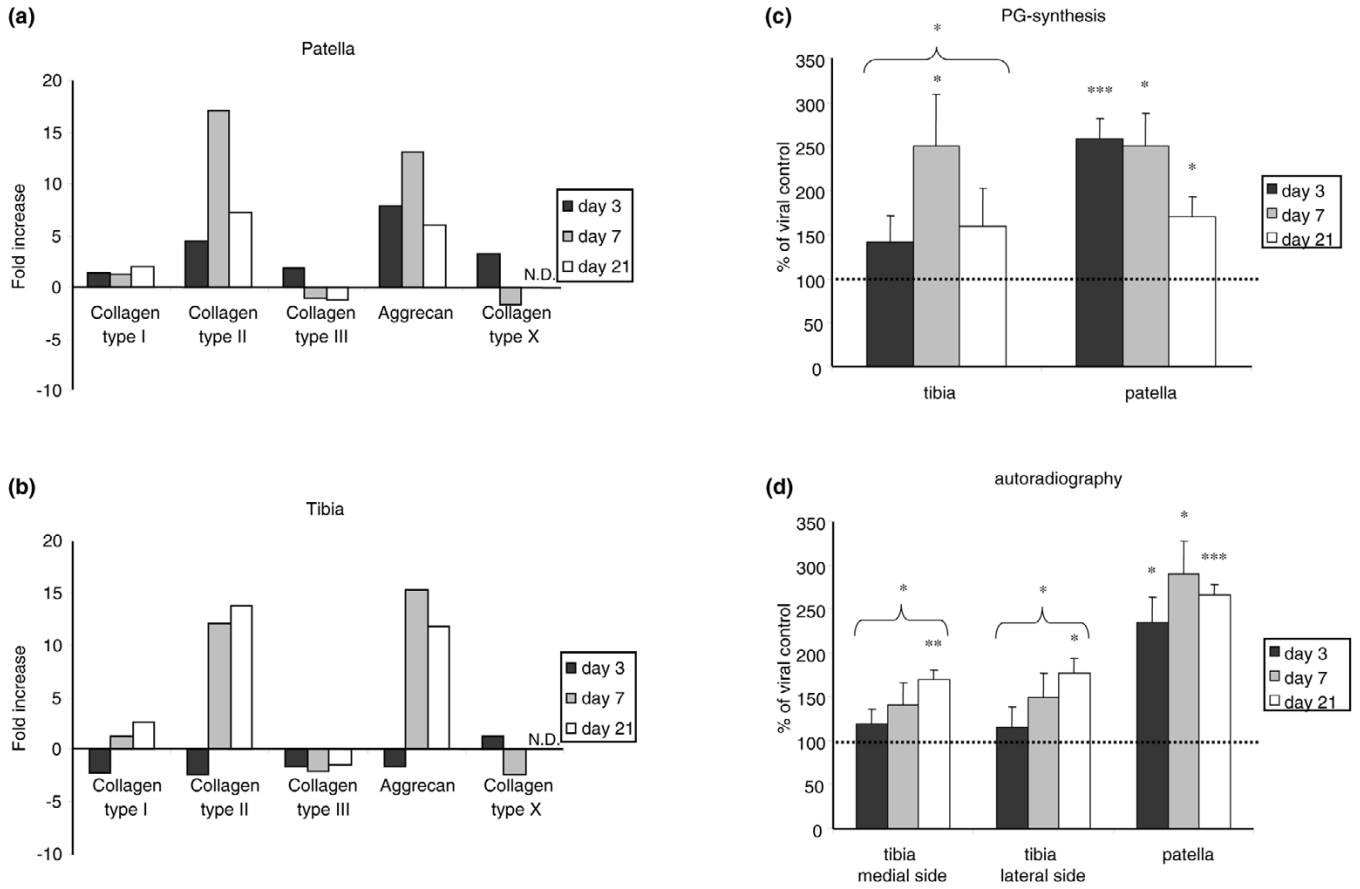
Effect of bone morphogenetic protein-2 (BMP-2) overexpression on mRNA levels of extracellular matrix molecules and proteoglycan (PG) synthesis. **(a,b) **Relative expression of mRNA levels of extracellular matrix molecules. Cartilage of mice injected with either Ad-BMP-2 or Ad-luc was isolated after 3, 7, and 21 days. Cartilage was pooled per group per time point, and RNA was isolated. Cycle threshold values were first corrected for *GAPDH *and then for the viral control, after which the fold increase/decrease was calculated. Decreases in mRNA levels compared with controls are on the negative scale. BMP-2 induced elevated levels of collagen type II and aggrecan. No changes in collagen type X expression were found. **(c,d) **Effect of BMP-2 overexpression on PG synthesis. Murine knee joints were injected with either Ad-BMP-2 or a control virus. Cartilage was isolated 3, 7, or 21 days after viral injection and incubated with ^35^SO_4_^2-^, after which the amount of incorporation was measured **(a)**. To perform autoradiography, mice were injected with ^35^SO_4_^2- ^intraperitoneally prior to knee joint isolation, which was performed 3, 7, or 21 days after viral injection. **(b) **These data show that BMP-2 stimulation of cartilage results in increased synthesis of PGs. Statistical analysis with a Student *t *test. *p < 0.05; **p < 0.005; ***p < 0.0005. N.D., not detectable.

On mRNA levels, Ad-BMP-2 transfection induced elevated expression of the extracellular matrix molecules collagen type II and aggrecan, in a similar magnitude on the tibia and the patella. Aggrecan mRNA was highest on day 7, with 13- and 15-fold increases on the patella and the tibia, respectively. On the patella, collagen type II mRNA had reached a 17-fold increase compared with controls (day 7). On the tibia, collagen type II had increased 12-fold on day 7 and was even higher by day 21 (14-fold increase compared with controls) (Figure 2a,b). In addition, mRNA levels of collagen type X were measured to investigate the possibility of chondrocyte hypertrophy because of the enlarged chondrocytes, but no differences between BMP-2-exposed cartilage and controls were found (Figure 2a,b).

### BMP-2 induces elevated proteoglycan synthesis

Elevated aggrecan expression was found on mRNA levels. To investigate whether this translated into an actual production of aggrecan, ^35^SO_4_^2- ^incorporation into patellar and tibial cartilage was assessed. The cartilage of the patella and the tibia was isolated 3, 7, and 21 days after viral injection and incubated with ^35^SO_4_^2- ^for 3 hours. The proteoglycan synthesis was found to be elevated in all cartilage surfaces. In tibial cartilage, ^35^SO_4_^2- ^incorporation had increased significantly on day 7, with 2.5-fold compared with Ad-luc controls. In patellar cartilage, the ^35^SO_4_^2- ^incorporation had reached 2.6-fold by day 3, 2.5-fold on day 7, and 1.7-fold by day 21 (Figure 2c).

In addition, to evaluate whether the increase in ^35^SO_4_^2- ^incorporation was distributed evenly in the cartilage or incorporated in a more focal fashion, autoradiography was performed. Therefore, mice were injected with ^35^SO_4_^2- ^prior to knee joint isolation. Autoradiography displayed the ^35^SO_4_^2- ^incorporated into the cartilage, which was distributed evenly along the chondrocytes in the non-calcified cartilage (Figure 1e–h). BMP-2 significantly increased proteoglycan synthesis in patellar cartilage on all days, up to almost 3-fold on day 7. The tibia also showed clear elevated proteoglycan synthesis upon stimulation with BMP-2, which had reached statistical significance by day 7. In patellar cartilage, the elevated proteoglycan synthesis seemed to have reached a plateau around day 7. In the cartilage of the tibia, ^35^SO_4_^2- ^incorporation increased with time (Figure 2d). There is a discrepancy between the data obtained with autoradiography and those obtained with *in vitro *^35^S incorporation. However, the data were collected in different experiments, and incubation periods and conditions were different (*in vivo *versus *in vitro*), which could have led to a difference in pattern. In both methods, a clear increase in proteoglycan synthesis was found.

### Proteoglycan content

**Figure 3:**
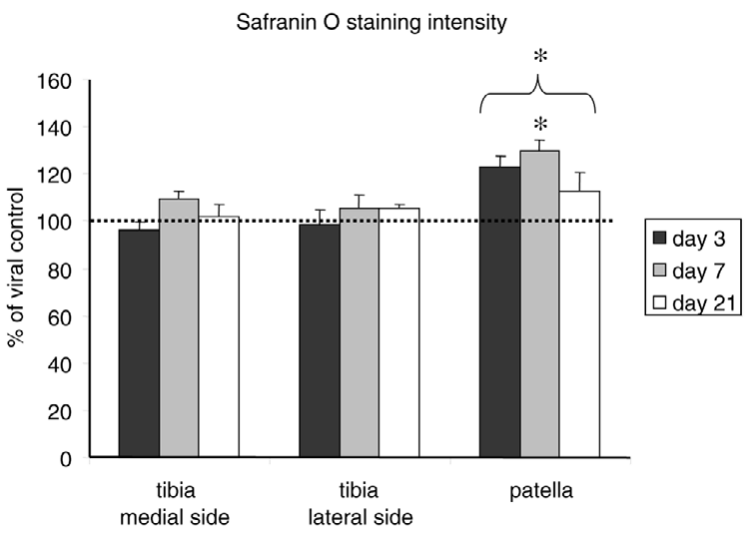
Proteoglycan content after Ad-BMP-2 injection. Knee joints of mice injected with Ad-BMP-2 or a control virus were isolated at days 3, 7, or 21 and processed for histology. Sections were stained with safranin O and fast green, after which safranin O staining intensity was measured in the articular cartilage with a computerized imaging system as a measurement of proteoglycan content of the cartilage. At least three sections per knee joint were measured. Measurements were averaged per knee joint. Statistical analysis with a Student *t *test. *p < 0.05. BMP-2, bone morphogenetic protein-2.

Although elevated levels of proteoglycan synthesis were found, no differences in safranin O staining intensity between BMP-2-exposed cartilage and controls were observed by mere visual investigation. Therefore, the safranin O staining intensity was scored in patellar and tibial cartilage with a computerized imaging system. There was a significant (30%) increase in safranin O staining intensity in the patella on day 7. When the data of all time points were pooled, a significant increase in safranin O staining was observed. The tibial cartilage, however, did not display any alterations in safranin O staining intensity (Figure 3). This is a discrepancy with the previously found elevated proteoglycan synthesis in the tibia, indicating that there might be additional degradation as well.

### MMP-mediated proteoglycan cleavage

**Figure 4:**
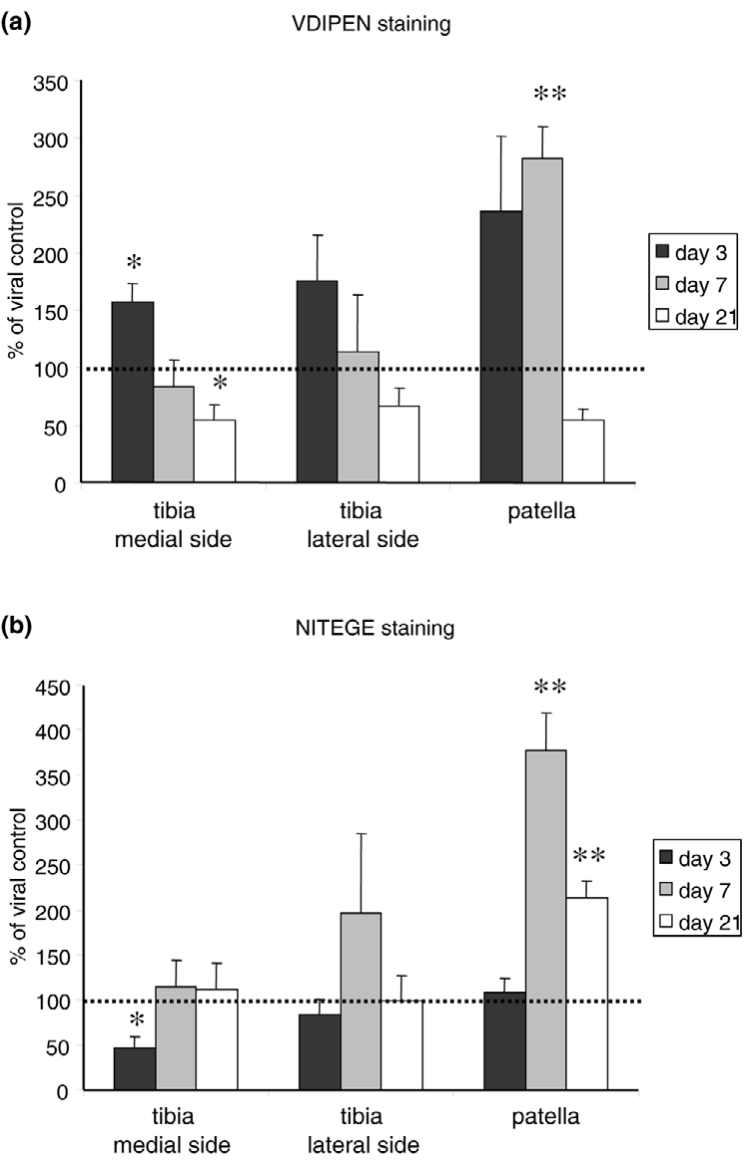
Effect of bone morphogenetic protein-2 (BMP-2) on cartilage matrix degradation. Immunohistochemistry for VDIPEN and NITEGE was performed on paraffin sections of knee joints 3, 7, and 21 days after Ad-BMP-2 or Ad-luc injection. The area of the cartilage staining positive was determined with a computerized imaging system. BMP-2 was compared to controls and shows an increase in VDIPEN staining on days 3 and 7 in patellar cartilage, which was only significant and most prominent on day 7. The elevated VDIPEN staining had reversed by day 21 to levels lower than those of controls. **(a) **NITEGE staining was low on day 3 but was clearly elevated by day 7 on the patella. This was reduced by more than 50% by day 21. **(b) **Statistical analysis with a Student *t *test. *p < 0.05; **p < 0.005.

To explore the possibility of elevated aggrecan degradation upon BMP-2 stimulation, paraffin sections of the knee joints were isolated on days 3, 7, and 21 after Ad-BMP-2 injection and were stained immunohistochemically for VDIPEN (Figure 1i–l). BMP-2 initially induced an increase in VDIPEN staining on day 7 in patellar cartilage. In tibial cartilage, elevated levels of VDIPEN staining were found on day 3. A significant decrease in VDIPEN staining was observed on the medial side of the tibia on day 21 (Figure 4a).

### ADAMTS-mediated proteoglycan cleavage

In addition to VDIPEN staining, NITEGE staining was performed (Figure 1m–p). NITEGE staining was lower than controls on day 3 in the cartilage on the medial side of the tibia, and no differences in the lateral tibial cartilage after BMP-2 stimulation were found. By day 7, NITEGE staining had increased in BMP-2-treated samples, with a significant 2.5-fold increase in the patella. By day 21, NITEGE staining was still significantly increased in the patella, but no differences in the tibia were found (Figure 4b).

### Role for BMP during natural reparative response upon damage

**Figure 5:**
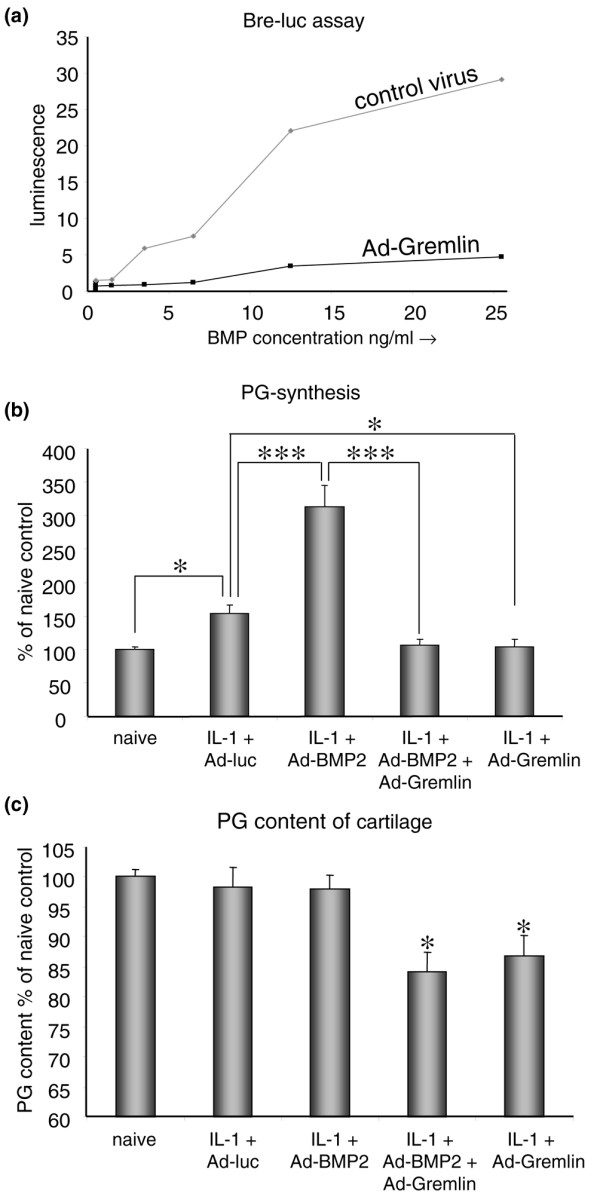
Role for bone morphogenetic protein (BMP) during natural reparative response to cartilage damage. To test whether the newly synthesized gremlin adenovirus was efficient in blocking BMP, 3T3 cells were transfected with Ad-gremlin or a control virus and the 28-hour supernatant was incubated with a variety of known concentrations of BMP-2 protein and the BRE-luciferase cell line. This cell line contains a luciferase construct coupled to a BMP-responsive element. Luminescence was measured and showed that Ad-gremlin blocked BMP-2 efficiently. **(a) **Mice were injected intra-articularly with interleukin-1 (IL-1)-beta to induce cartilage damage. After 2 days, an adenovirus expressing BMP-2, BMP-2 + gremlin, or a control virus was injected. After 4 days, patellae were isolated and incubated in medium with ^35^SO_4_^2- ^to assess proteoglycan (PG) synthesis **(b)**, or whole knee joints were isolated to measure PG content of the cartilage **(c)**. This showed that BMP-2 boosts PG synthesis and that blocking of BMP activity results in an abrogation of the natural reparative response after cartilage damage. **(b) **Moreover, blocking of BMP activity with gremlin resulted in an overall outcome of PG depletion. **(c) **Ad-gremlin injection alone, without IL-1, has no effect (data not shown). Statistical analysis with a Student *t *test. *p < 0.05; **p < 0.005; ***p < 0.0005. Bre-luc, bone morphogenetic protein-responsive element-luciferase.

To assess whether BMP signalling is required for cartilage repair and whether BMP activity can enhance cartilage repair, cartilage damage was induced with IL-1. Thereafter, either BMP activity was blocked or BMP-2 was added to see whether this influenced the natural reparative response. *In vivo *exposure of cartilage to IL-1 initially resulted in a decrease in proteoglycan synthesis. Thereafter, the synthesis levels quickly elevate even beyond normal turnover levels (overshoot). This can be observed first around day 4 after a single IL-1 injection [21]. On day 4 after IL-1 injection, proteoglycan synthesis was significantly increased: 53% greater than that in cartilage of control knee joints (overshoot) (Figure 5b). Overexpression of BMP-2 with an adenovirus gave rise to an increase in proteoglycan synthesis to more than 300% compared with normal turnover proteoglycan synthesis. To investigate the role of endogenous BMP in cartilage repair, BMP activity was inhibited by adenoviral overexpression of the BMP-inhibitor gremlin. The adenovirus overexpressing gremlin was found to efficiently block BMP activity (Figure 5a). Gremlin expression not only was able to totally abolish the boost in proteoglycan synthesis that was induced by BMP-2, but restrained the IL-1-related elevation in proteoglycan synthesis (Figure 5b).

To investigate the influence of the various conditions on the total proteoglycan content, the staining intensity of safranin O was measured in the cartilage. The damage that had been inflicted by IL-1 had been overcome by the natural repair of chondrocytes by day 4 (Figure 5c). Although BMP-2 induced an increase in proteoglycan synthesis, the outcome in proteoglycan content was comparable to the natural reparative response. However, when BMP activity was blocked by gremlin, the natural reparative response was abolished and resulted in proteoglycan depletion of the cartilage. These data show not only that BMP-2 is able to boost proteoglycan synthesis in damaged cartilage, but also that BMP plays a role in the natural reparative response of chondrocytes as a reaction to damage.

## Discussion

In the literature, BMP-2 is proposed as a stimulant for cartilage (re)generation. BMP-2 is able to stimulate proteoglycan synthesis in murine cartilage and enhances collagen type II expression in chondrocytes seeded in alginate [22,23]. Also, in species like rats and (most important) humans, BMP-2 is able to stimulate the chondrogenic phenotype on the mRNA level and to stimulate cartilage extracellular matrix proteoglycan production [24,25]. In this study, BMP-2 induced an increase in mRNA levels of collagen type II and aggrecan and stimulated proteoglycan synthesis up to three-fold *in vivo*, both in healthy and in damaged cartilage. All these data, including current data, confirm a strong anabolic effect of BMP-2 on cartilage.

What most studies neglect to investigate is whether there is a catabolic effect of BMP on intact cartilage. Indeed, BMP-2 exposure led to degradation of aggrecan as shown by the increase in MMP- and ADAMTS (a disintegrin and metalloproteinase with thrombospondin motifs)-mediated proteoglycan degradation. This is not necessarily negative for cartilage integrity, especially if the use of BMP-2 is intended as a stimulant of cartilage repair. In that case, it is not unlikely that old tissue has to be removed in order to provide space for the large amounts of newly synthesized extracellular matrix. The catabolic effects that were observed were temporary, as the evidence for MMP-mediated degradation was totally reversed by day 21 to levels lower than in control cartilage. ADAMTS-mediated degradation lingered but had also been reduced more than 50% compared with day 7. The degradational response might be the initial impulse of the chondrocytes to create space in the cartilage for the new tissue that will be generated. However, for BMP-2 to have a reparative effect, it is crucial that the degradational properties not exceed the production of extracellular matrix. Overall, BMP-2 increased proteoglycan content in patellar cartilage, showing that although there was degradational activity, BMP-2 had an overall anabolic effect.

The cartilage surfaces that were measured responded differently in the magnitude of their response. The conformation of the cartilage is likely to be different, as their weight-bearing properties require different stiffness of the cartilage. This might also influence the properties of the chondrocytes in the cartilage, hence their response to a stimulus. However, since the nature of the response is the same, this indicates that the response that was found is predictive for different cartilage surfaces.

Although the overall effect was anabolic, an altered appearance of the chondrocytes was observed, which was expected to be an alteration toward a hypertrophic state. On PCR levels, no upregulation of collagen type X was found, nor an upregulation in MMP-13 expression. This indicates that the altered appearance is not a hallmark of terminal differentiation. Therefore, the possibility of an altered proliferation rate causing the altered appearance was explored, potentially causing the cartilage to appear more cellular. Immunohistochemical staining for proliferating cell nuclear antigen showed no difference between BMP-2 and controls, resulting in dismissal of this theory (data not shown). The chondrocytes displayed a highly increased proteoglycan production but also a high degree of degradational activity. VDIPEN staining and NITEGE staining were particularly intense in the pericellular area surrounding the unusually large chondrocytes. It could be speculated that the partial degradation of the pericellular matrix, in combination with chondrocyte activation, could have given the impression of cell enlargement.

BMPs are growth factors that are necessary for cartilage formation during embryonic skeletal development [26]. The lack of BMP signalling in mice will result in a loss of cartilage as it wears away in BMP-receptor-1a-deficient mice. These data show that BMP is necessary for cartilage maintenance [27]. Besides cartilage maintenance, BMP-2 is beneficial for cartilage repair. This has been demonstrated by the fact that BMP-2 stimulates cartilage repair in defects filled with collagen sponges [28]. In addition, the use of rh-BMP-2 in full-thickness defects improves the properties of the newly synthesized cartilage [29]. Our group previously found that BMP-2 was low in healthy cartilage but was expressed in areas surrounding cartilage damage or in osteoarthritic cartilage in mice [12]. Nakase and colleagues [30] found a similar localization in humans. This indicates that BMP-2 is upregulated in injured areas. BMP upregulation was also found in other kinds of injury such as in mechanically injured cartilage explants but also in chondrocytes stimulated with either IL-1 or tumor necrosis factor-alpha (TNF-α) [13,31]. In this study, the importance of BMP for cartilage repair was the confirmed blocking of BMP activity after IL-1-induced cartilage damage resulted in an abrogation of the natural reparative response by chondrocytes. These data confirm those of Fukui and colleagues [32], who demonstrated a similar effect in chondrocytes exposed to TNF-α. When BMP activity was blocked by noggin during TNF-α exposure, the proteoglycan synthesis was reduced. BMP-2 is apparently necessary for cartilage integrity and improves its repair.

Our group has previously shown that, like BMP, TGF-β increased proteoglycan synthesis and that blocking of TGF-β, much like blocking of BMPs as shown in the present paper, abolished the proteoglycan overshoot after IL-1 induced cartilage damage [33]. Blocking either TGF-β or BMP is apparently sufficient to block the natural reparative response. This indicates that intrinsic TGF-β and BMP act synergistically in IL-damaged articular cartilage. During experimental OA, TGF-β signalling decreases whereas BMP-2 expression is induced [12]. Taking into account the present data, one could speculate that the increase in BMP-2 is a means of compensation for the lack of TGF-β and thus a functional response to injury. The eventual cartilage loss observed in OA shows that BMP activity alone is not sufficient to adequately protect cartilage against destruction.

Overall, these findings imply that the expression of BMP in OA cartilage is an anabolic response to injury in an attempt of the chondrocytes to compensate for the catabolic effects of both cytokine-induced and mechanically induced injury. The BMP-2-induced elevated degradational activity is most likely an attempt to clear away old matrix molecules to make room for the newly synthesized molecules, indicating a role for BMP-2 in cartilage remodeling. Alternatively, it can be that the newly synthesized aggrecan molecules are more vulnerable to degradation, leading to the increased presence of VDIPEN and NITEGE epitopes in the BMP-2-exposed cartilage.

## Conclusion

These data show that BMP-2 exposure resulted in a strong stimulation of proteoglycan synthesis, both in healthy and in damaged cartilage. Blocking endogenous BMP activity compromised cartilage repair. Moreover, BMP-2 clearly elevated degradation of aggrecan, mediated by MMPs and ADAMTS. Thus, BMP activity appears to be involved in cartilage repair and the replacement of damaged matrix molecules.

## Abbreviations

ADAMTS = a disintegrin and metalloproteinase with thrombospondin motifs; BMP = bone morphogenetic protein; BRE = bone morphogenetic protein-responsive element; CMV = cytomegalovirus; Ct = cycle threshold; IL-1 = interleukin-1; MMP = matrix metalloproteinase; MOI = multiplicity of infection; OA = osteoarthritis; PBS = phosphate-buffered saline; PCR = polymerase chain reaction; PFU = plaque-forming unit; Q-PCR = quantitative polymerase chain reaction; TGF-β = transforming growth factor-beta; TNF-α = tumor necrosis factor-alpha.

## Competing interests

The authors declare that they have no competing interests.

## Authors' contributions

EBD participated in conceiving this study, designed this study, designed and constructed the gremlin adenovirus, participated in the animal experiments, participated in histology, performed the NITEGE immunohistochemistry, performed all histological scores, analyzed the data, and drafted the manuscript. EV constructed the stable cell line containing the obtained BRE-luciferase construct, performed the BRE-luciferase measurements, participated in the animal experiments, carried out histological processing of the knee joints, participated in the histology, performed the autoradiography, and performed measurement of ^35^SO_4_^2- ^incorporation. PvL participated in the VDIPEN immunohistochemistry. FvdL supervised and designed the construction of the BMP-2 adenovirus. PvdK conceived of the study, participated in the design and coordination, and helped to draft the manuscript. WvdB participated in study design and revision of the final manuscript. All authors read and approved the final manuscript.
